# Deep learning based automation of mean linear intercept quantification in COPD research

**DOI:** 10.3389/fdata.2025.1461016

**Published:** 2025-06-10

**Authors:** Lars Leyendecker, Anna Louisa Weltin, Florian Nienhaus, Michaela Matthey, Bastian Nießing, Daniela Wenzel, Robert H. Schmitt

**Affiliations:** ^1^Department of Production Quality, Production Metrology and Bio-Adaptive Production, Fraunhofer Institute for Production Technology IPT, Aachen, Germany; ^2^Department of Systems Physiology, Medical Faculty, Ruhr University of Bochum, Bochum, Germany; ^3^Medical Faculty, Institute of Physiology I, University of Bonn, Bonn, Germany; ^4^Intelligence in Quality Sensing, Laboratory for Machine Tools and Production Engineering WZL of RWTH Aachen, Aachen, Germany

**Keywords:** deep learning, pulmonary disease, mean linear intercept (MLI), semantic segmentation, microscopy, automated machine learning

## Abstract

Chronic obstructive pulmonary disease (COPD), a major cause of global mortality, necessitates novel therapies targeting lung function and remodeling. Their effect on emphysema formation is initially investigated using mouse models by analyzing histological lung sections. The extent of airspace enlargement that is characteristic for emphysema is quantified by manual assessment of the mean linear intercept (MLI) across multiple histological microscopy images. Besides being tedious and cost intensive, this manual task lacks scientific comparability due to complexity and subjectivity. In order to continue with the well-established practice and to preserve the comparability of study results, we propose a deep learning-based approach for automating the determination of MLI in histological lung sections utilizing the AutoML software *AIxCell* which is specialized for the domain of semantic segmentation-based cell culture and tissue analysis. We develop and evaluate our image processing pipeline on stained histological microscope images that stem from a study including two groups of C57BL/6 mice where one group was exposed to cigarette smoke while the control group was not. The results indicate that the *AIxCell* segmentation algorithm achieves excellent performance, with IoU scores consistently exceeding 90%. Furthermore, the automated approach consistently yields higher MLI values compared to the manually generated values. However, the consistent nature of this discrepancy suggests that the automated approach can be reliably employed without any limitations. Moreover, it demonstrates statistical significance in distinguishing between smoker's and non-smoker's lungs.

## 1 Introduction

Chronic obstructive pulmonary disease (COPD) is the third leading cause of death worldwide and its prevalence is still increasing (Li et al., 2023). Besides cigarette smoking, environmental and occupational factors are common causes of COPD (Holtjer et al., 2023). The disease is characterized by inflammation and remodeling of lung tissue (Jeffery, 2001). Long-term remodeling can result in destruction of alveoli and airspace enlargement, which are characteristic features of emphysema (Wang et al., 2018).

Because current therapies only have a limited effect on lung remodeling and emphysema, new molecular targets and pharmacological compounds are urgently needed (Lo Bello et al., 2020). To analyze the effect of potential new therapies on COPD, the utilization of animal models in general and mouse models in particular is very valuable because they reflect the complexity of a whole organism including all cell types and tissues. The mouse model with the highest pathophysiological relevance for COPD is chronic cigarette smoke exposure (Ghorani et al., 2017). After several months of smoking, lungs are harvested and remodeling and emphysema formation are assessed by analyzing histological lung sections (Yao et al., 2010).

The mean linear intercept (MLI) method is commonly used to assess emphysema by quantifying the chord length in microscope images of stained lung sections. Test lines of known length are overlaid on lung images, and intersections with the alveolar surface are counted, inversely correlating with the mean linear intercept chord length (*L*_*m*_) (Knudsen et al., 2010). This analysis is still performed manually by specialists being highly labor- and cost-intensive, with evaluation complexity and observer-dependency reducing result comparability. In recent years, image processing and recognition algorithms along with Deep Learning (DL) methods utilizing artificial neural networks have proven to be effective for automating and objectivizing visual recognition tasks by extracting semantic information from high-dimensional microscopy image data (Tsuneki, 2022; Fuyong et al., 2018). However, a reliable implementation, training and maintenance of such DL pipelines requires various skills from the fields of software engineering, programming, data analysis, and DL along with sufficient computational resources for training and inferencing (Jain et al., 2024; Stoean et al., 2023). The majority of biological and medical experts lack these skills hindering them to utilize the technological potential for their individual analyses.

To automatize analysis with minimal configuration and customization effort and to promote the adaptation of DL methods in biomedical practice, we have developed *AIxCell*—an image-based automated machine learning (AutoML) system that is tailor-made for the domain of cell culture analysis (Leyendecker et al., 2022; Baratchi et al., 2024). Given its domain-specificity, we claim *AIxCell* to be a domain-specific AutoML-system that incorporates a portfolio-based meta-learning approach for automating ML development for a provided labeled dataset. It's meta learning model aims to configure a best-performing DL pipeline including pre-processing and post-processing steps for a given analysis task and image data. To do so, it utilizes knowledge from a portfolio of previously solved related analyses represented as in meta data. This meta data contains information on the analysis task, the dataset, the DL pipeline configuration and final evaluation results. To force its adoption in biomedical practice, *AIxCell* provides a user-friendly interface that guides the user through the different steps of analysis configuration, image annotation and semantic segmentation-based image processing and evaluation (Leyendecker et al., 2022). To both automate the quantification of emphysema formation and to extend the AutoML system from cell culture analysis to tissue analysis we aim to solve the pulmonary disease use-case using *AIxCell*.

This paper presents the medical background of pulmonary alveoli analysis and currently used manual methods. Subsequently, we introduce our DL-based approach by discussing the image processing pipeline. The main contributions of our paper are:

- Developing a modular Deep Learning pipeline for the automation of mean linear intercept (MLI) quantification.
- Presenting another use-case of our domain-specific Auto-ML system *AIxCell* Leyendecker et al. (2022) on automating biomedical analysis on microscopy images of histological lung section.
- Demonstrating that *AIxCell* is capable of accurately segmenting microscopy images of pulmonary alveoli improving the quality of biomedical analysis in comparison to manual analyses.

To evaluate the effectiveness of our approach, tests are conducted to compare the DL method with the manual approach. Lastly, the application of the proposed method is discussed in the context of biological relevance. The following sections detail each step of this investigation in detail.

## 2 Materials and methods

The aim of this study was to automize the measurement of pulmonary airspace enlargement. We begin by presenting the biological study, followed by a description of the manual analysis method. Subsequently, we introduce our DL-based approach utilizing the domain-specific AutoML-system *AIxCell*. We then proceed to outline the evaluation of our new approach and its comparison with the manual method.

### 2.1 Smoke exposure and manual analysis of lung sections

The following section describes the study design used to determine pulmonary airspace enlargement manually.

#### 2.1.1 Biological experiment design

Animal experiments were performed according to the guidelines of the German law of protection of animal life with approval by the local government authorities (LANUV, NRW, Germany). Even though there are several animal models of COPD, e.g., based on lipopolysaccharide or elastase instillation into the lung, the current gold standard is cigarette smoke-induced COPD, as cigarette smoke is the major risk factor for COPD in humans (Churg et al., 2008) also rats, guinea pigs and dogs are used, the most commonly applied species is mouse, as it enables the use of knockout animals and there are many molecular tools available (Ghorani et al., 2017).

To cause cigarette smoke-induced chronic obstructive pulmonary disease (COPD) in female C57BL/6 mice, we utilized a modified version of the protocol developed by de Souza et al. (2014). The “in Expose” model (Scireq, Montreal, Canada) was employed for this purpose. Mice were exposed to the cigarette smoke of 3R4F research cigarettes (University of Kentucky, Lexington, USA). The exposure to mainstream cigarette smoke occurred twice daily, five days a week, over a period of 20 weeks to induce COPD. During each exposure session of 1 h, smoke was generated from 24 cigarettes at a rate of 4 puffs per minute (*n* = 3). Control mice were exposed to room air (*n* = 3).

At the end of the protocol, lung function was determined by a flexiVent system (Scireq). Therefore, mice were anesthetized with fentanyl (50 μg/kg), medetomidine (0.5 mg/kg), and midazolam (5 mg/kg). Then, they were tracheally cannulated and ventilated with a tidal volume of 10 ml/kg at a frequency of 150 breaths/min and a positive end-expiratory pressure (PEEP) of 2.5 cmH_2_,O.

To analyze the increase in airway reactivity, escalating doses of methacholine were administered as an aerosol using a nebulizer (0, 6.25, 12.5, 25, and 50 mg/ml, 25 μl each). Additionally, pressure-volume loops were recorded. At the end of the experiment animals were euthanized by cervical dislocation and lungs were removed.

#### 2.1.2 Image generation of lung sections

For histological analysis, the lungs were perfused with 4% paraformaldehyde (PFA) at a pressure of 25 cmH_2_O. Following overnight fixation, paraffin sections were prepared, and subsequent H&E staining was performed. Images were captured using an Axiostar Plus microscope equipped with an Axiocam MRc5 camera, a 20× A-Plan objective (NA: 0.45) and the Axiovision software (Zeiss, Germany).

To calculate the mean linear intercept (MLI), 12 randomly selected fields from different lobes of the right lung were analyzed for each mouse. Images containing large vessels and airways were excluded from the analysis.

#### 2.1.3 Mean linear intercept

Mean linear intercept (MLI) is a common method to estimate the respiratory volume-to-surface ratio of lung tissue (WEIBEL and GOMEZ, 1962). There is a range of different implementations and versions, both manual and automatised, with different strengths and weaknesses (Knudsen et al., 2010) [MBCA+12] (Parameswaran et al., 2006). Our study implements the following approach: An arbitrary number of lines is drawn through the lung image. The total length of these lines is denoted as *l*. Each time a line crosses the border from background to tissue or vice versa, an intercept is counted. The total number of intercepts is then denoted as *n*. The mean linear intercept *L*_*m*_ can then be computed according to the following formula:


(1) $$\begin{array}{l} L_{m}=l/n \end{array}$$

**Figure 1:**
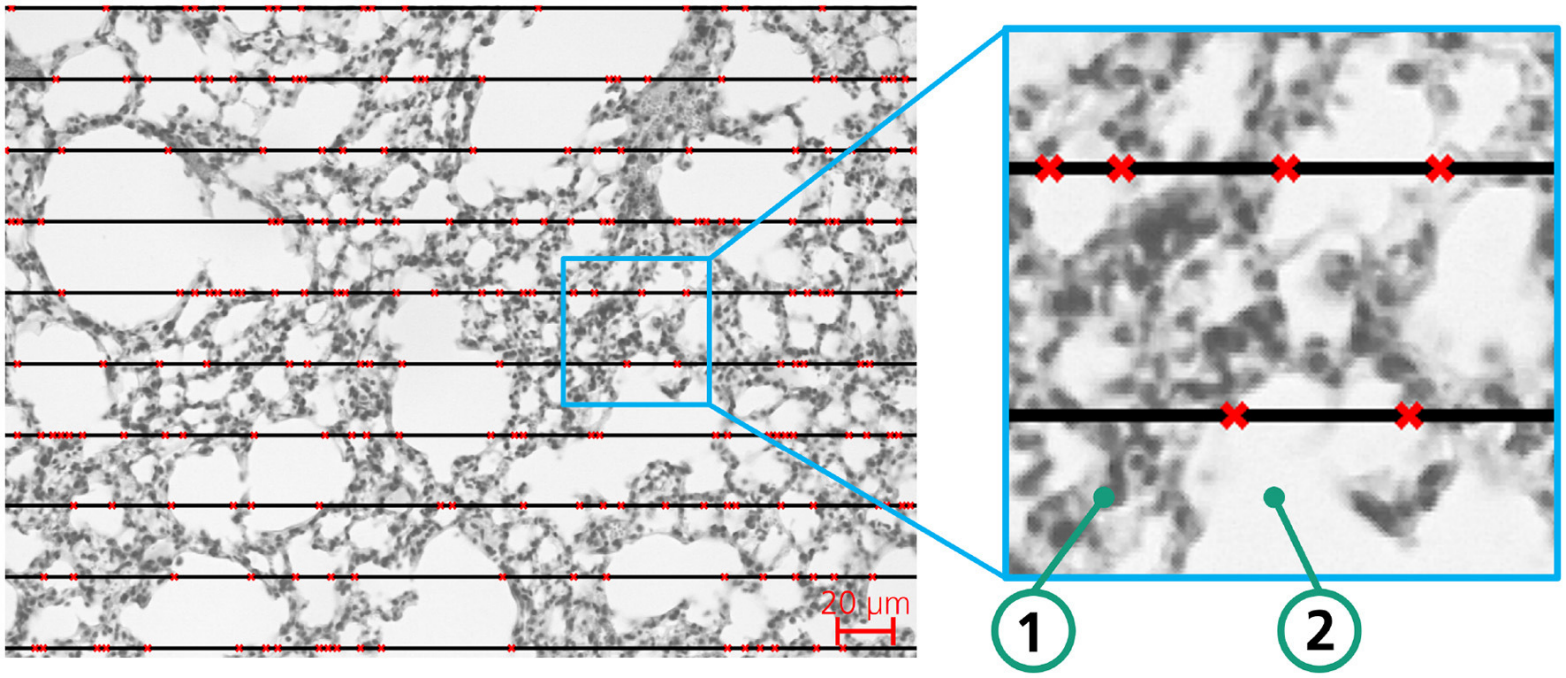
Exemplary lung tissue sample of a mouse to illustrate the MLI method for determination of airspace enlargement; red crosses mark the transition between background (white) and tissue.

To make different images comparable, we used ten equidistant lines throughout all images. One example image is shown in Figure 1. *L*_*m*_ is not a direct measure of alveolar size as also alveolar ducts are comprised in sections of lung parenchyma. In addition, it is dependent on the degree of lung inflation and therefore rather reflects the surface to volume ratio of acinar airspaces in general (Hsia et al., 2010; Knudsen et al., 2010). Still, *L*_*m*_ is considered as an accepted measure of airspace enlargement of emphysema.

### 2.2 DL based analysis approach utilizing AIxCell, a domain-specific AutoML-system for cell culture analysis

**Figure 2:**
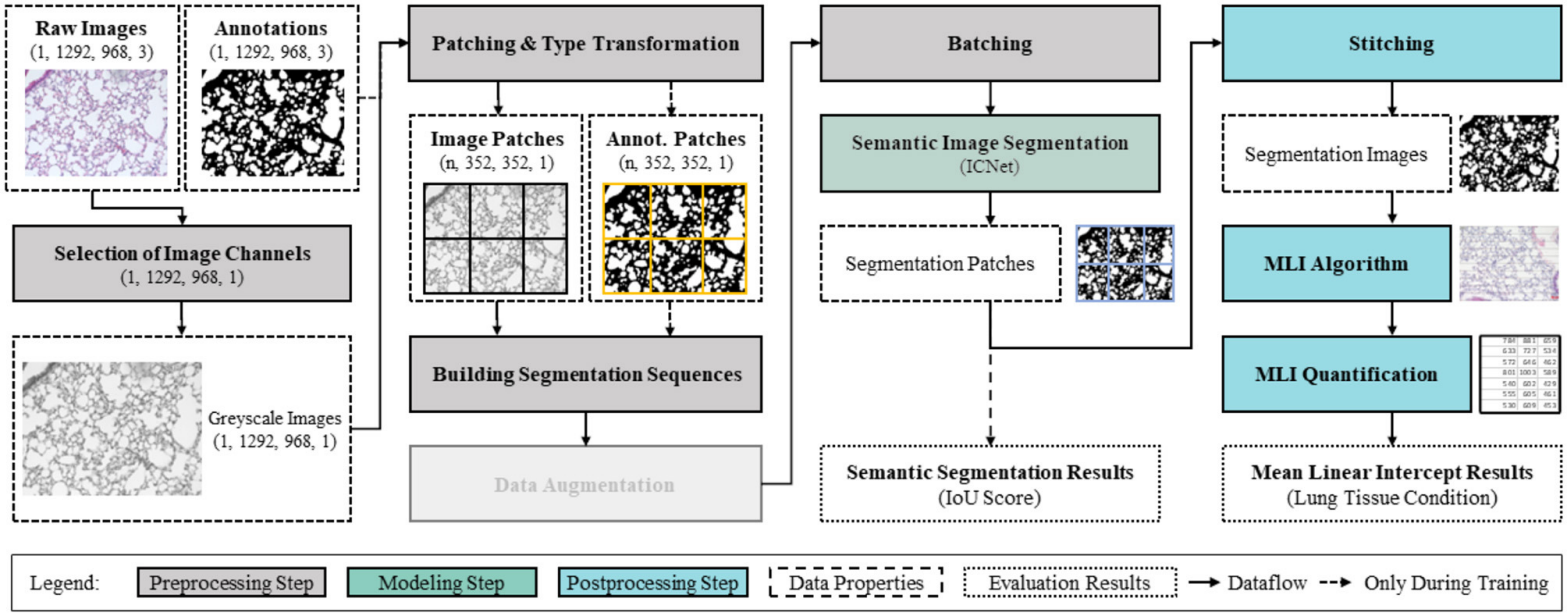
Modular image analysis pipeline constituted of pre-processing, modeling and post-processing with corresponding data properties.

*AIxCell* is a domain-specific AutoML-System for automating microscopy image-based cell and tissue analyses. Thereby, we aim to enable biomedical experts to utilize the image analysis capabilities of deep convolutional neural networks and machine learning algorithms without requiring DL or programming expertise. *AIxCell* consists of a modular DL-based pipeline structure that, according to Figure 2, is trisect into pre-processing, modeling and post-processing. *AIxCell* enables biomedical experts to utilize DL-based image recognition for automating their individual microscopy datasets and analysis tasks. In addition to pre-configured pre-processing and post-processing steps, its modular design also enables the implementation of application-specific procedures introducing a considerable level of flexibility (Leyendecker et al., 2022). In its core component, *AIxCell* utilizes convolutional deep neural networks (CNN) and an encoder-decoder architecture to perform supervised semantic segmentation for arbitrary image feature extraction. As with the majority of data-driven systems, both performance and applicability of *AIxCell* scale with the number of datasets and use-cases that were realized with it. To automate MLI analysis in COPD research, we extend *AIxCell's* module library that stores of all its pre-processing, modeling, and post-processing functionality by implementing use-case specific post-processing modules for MLI calculation. Therein, data pre-processing, semantic segmentation, computation of the MLI and quantification are sequentially performed to determine the MLI and therefore the extent of airspace enlargement. We then apply *AIxCell* to the lung image dataset to provide a fully-functional image analysis pipeline for this use-case and to demonstrate the applicability and functionality of *AIxCell* on another use-case.

Our final image image analysis pipeline for MLI quantification in microscopy images is shown in Figure 2. For both training and inference, the pipeline comprises all image processing steps and data flows beginning with the raw microscopy images and and ending with the MLI results. In the following, starting with describing dataset properties (Section 2.3), we further outline the overall configuration of this pipeline comprising pre-processing (Section 2.3.1), DL-based modeling (Section 2.3.2), and Post-processing and MLI quantification (Section 2.3.3). In Section 2.3.4, we define the performance metrics and outline the training procedure.

### 2.3 Dataset properties of lung images

**Figure 3:**
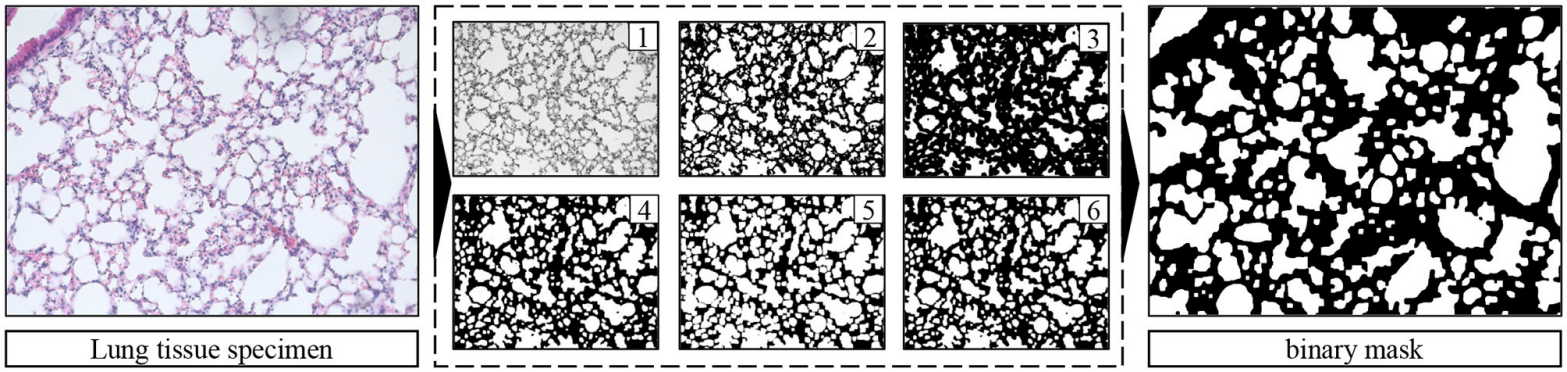
Raw image **(left)**, binary semantic segmentation mask **(right)**, and corresponding threshold-based image conversion procedure for automated image annotation (steps 1–6).

The raw microscopy image dataset comprises 72 images of histological sections of lung tissue. Each image has a dimensionality of 1,292 × 968 pixel and three color channels. As required for supervised learning, we have implemented a threshold-based image conversion procedure for automatically creating semantic segmentation annotations. This procedure comprising greyscale conversion (step 1), thresholding (step 2), white erosion (step 3), white dilation (steps 4 and 5), and a final white erosion operation (step 6) is outlined in Figure 3.

#### 2.3.1 Pre-processing

According to the data-pipeline (see Figure 2), the raw microscopy images from the lung image dataset are preprocessed and enhanced to achieve optimal modeling and subsequent analysis results. First, due to the need for dimensionality reduction, the raw images of dimensions (1,292, 968) comprising three color channels are converted to greyscale. Thereby, the computational efficiency of the data pipeline is enhanced. We design all subsequent operations to be conditional on these dimensions, so that the pipeline can cope with varying image dimensions. For further reducing the required size of the convolutional neural network and thereby training and inference times, both images and annotations are equally split into patches of subordinate size. To avoid information loss in near patch border regions, the images are patched using a stride of half the patch dimensions (352*px*/2 = 176*px*) producing overlapping patches. The resulting image patches have the dimensions (1, 352, 352, 2). In preliminary experiments, these patching dimensions have been identified in maximizing F1-Score and minimizing both validation loss duration of a single training epoch. Thereby, no informational content is lost due to dimensionality reduction while an efficient neural network size can be maintained. After sequencing the cached patches for efficient memory usage, they are forwared to the semantic segmentation model for training in batch-mode. Please note that in contrast to the use-case presented in Leyendecker et al. (2022), for this use-case, no oversampling and image augmentation techniques are applied.

#### 2.3.2 Modeling

In the modeling step, we use a symmetrical convolutional encoder-decoder network architecture for pixelwise semantic segmentation and choose intersection over union (IoU) and F1-score metrics for evaluating the performance of the segmentation model (see Section 2.3.4) (Minaee et al., 2022). Given the modular structure of the image analysis pipeline, we utilize semantic segmentation because, in comparison to end-to-end image analysis pipelines, it provides the most flexibility in combination with use-case specific post-processing procedures. Thereby, we achieve a high degree in flexibility and therefore applicability of *AIxCell* to a variety of different cell culture analysis use-cases. In general, by using semantic segmentation, one aims to identify and group pixels that share similar properties thereby reducing the an image pixel variety to the number of pre-defined segmentation classes (Szeliski, 2022). Two main types of image segmentation methods exist: semantic segmentation and instance segmentation. Semantic segmentation classifies individual pixels into distinct classes, while instance segmentation also identifies separate objects within the same class (Szeliski, 2022). Because in the majority of cell and tissue analysis tasks considering single cell information does not provide relevant information and considering the increased labeling efforts for instance segmentation, we decided to incorporate semantic segmentation instead of instance segmentation into *AIxCell*. For the COPD use-case in particular, an instance-based approach is also not suitable because the separation of individual alveoli does not yield important information for MLI quantification.

Given the symmetrical shape of encoder-decoder network architectures, both input and output layer of the network are of equal dimensions. By enforcing the input data into a lower-dimensional representation in the bottleneck of the network and subsequently increasing the dimensions of the data in the decoder, the model provides an output of same dimension as the input data only containing the features of interest in form of pixelwise classification. For processing two-dimensional microscopy images that have been converted to grayscale in the pre-processing procedure, we utilize networks with two-dimensional convolutional layers. Suitable network architectures comprise U-Net (Ronneberger et al., 2015), Feature Pyramid Network (Seferbekov et al., 2018), LinkNet (Chaurasia and Culurciello, 2017), ICNet (Zhao et al., 2018), and PSPNet (Zhao et al., 2017). Based on preliminary experiments, we selected and configured the ICNet architecture.

#### 2.3.3 Post-processing

In post-processing, the patches are stitched back together to obtain the dimension of the input image. Based on the segmentation image, post-processing functions aim to extract analysis-specific information. For the MLI computation that resembles the manual process and, therefore, ensures comparability of scientific results, we have developed a custom algorithm. We draw a pre-configurable number of horizontal lines (compare with Figure 3 left) as an additional layer on top of the binary segmentation mask. The algorithm then sequentially follows each line, checking whether the pixel above or below the line is facing a color swap. If so, the interception counter *n* increases by one. To avoid overcounting of intercepts, we introduce a regularization hyperparameter (determined in a series of tests with subsequent evaluation by biomedical experts) that specifies how many pixels after a detected change between tissue and background must be passed before a new change can be detected. If all horizontal lines have been traversed, the MLI is calculated according to Equation 1.

#### 2.3.4 Training procedure and performance metrics - F1-Score and Intersection Over Union (IoU)

For training the CNN encoder-decoder architecture, we split the total of 72 images into three distinct datasets for training, validation, and testing. After performing the split, the training set contains 50 images, and both validation and testing sets contain 11 images each. We trained the ICNet architecture for 20 epochs using a learning rate of 0.0001 (default case) and a batch-size of 9 (determined in preliminary experiments). As evaluation metrics to assess the accuracy of semantic image segmentation, we apply two types of metrics, namely intersection over union (IoU score) and F1 score (Minaee et al., 2022). The IoU-score is a common evaluation metric for semantic segmentation comparing the similarity of two sets A and B (See Equation 2). For the binary classification case, the IoU-score can be simplified as the ratio of *true positives (TP)* and the sum of *true positives (TP), false negatives (FN)*, and *false positives (FP)*. The IoU score is also known as the Jaccard index *J*(*A*; *B*) (Rezatofighi et al., 2019). In the context of ML, it represents the overlap between the prediction and the ground truth of a class divided by the union between the prediction and ground truth. The IoU-score thereby quantifies how accurate the semantic segmentation model is in distinguishing objects from the image background and allows to calculate the segmentation performance for distinct classes separately.


(2) $$\begin{array}{l} IoU=\frac{\left|A\cap B\right|}{\left|A|\cup |B\right|} \end{array}$$

(3) $$\begin{array}{l} IoU=\frac{TP}{TP+FN+FP} \end{array}$$

According to Equation 6, the F1-score represents the harmonic mean of precision (see Equation 4) and recall (see Equation 5) for a given class *k*. Both metrics share the same value range between 0 and 1, whereas 1 indicates a perfect match between the segmentation mask and ground truth, while 0 describes their perfect mismatch.


(4) $$\begin{array}{l} precision_{k}=\frac{TP_{k}}{TP_{k}+FP_{k}} \end{array}$$

(5) $$\begin{array}{l} recall_{k}=\frac{TP_{k}}{TP_{k}+FN_{k}} \end{array}$$

(6) $$\begin{array}{l} F1_{k}=\frac{2\;\cdot \;precision_{k}\;\cdot \;recall_{k}}{precision_{k}+recall_{k}} \end{array}$$

### 2.4 Evaluation

To evaluate the potential of replacing the manual method with the DL-based MLI approach, three tests were conducted. In the first test, we quantified the training performance of the neural network. The data set was divided into three sets of equal size: training, validation, and testing. Each set contained images of all six mice. IoU-values and F1-scores were calculated for the evaluated images using the automatically labeled images as reference. Average F1 and IoU scores were then determined for each dataset.

Next, the second test involved calculating the MLI using three different methods on the same dataset. These methods included manual labeling and counting, classic image segmentation (the one used for generating training data), and DL segmentation. For both automated segmentation approaches, the same algorithm was employed to calculate the number of intersects based on the segmented images. The MLI scores were averaged for smokers and non-smokers separately, resulting in average MLI values and corresponding standard deviations for each group. These values were then used to compare the different approaches.

In the third test, hypothesis tests were conducted to determine whether there were significant differences in MLI values within the groups. This analysis was performed for both manually and DL-based calculated MLI values. The Student-t-test was chosen as the statistical test since a standard deviation could be assumed within each dataset (smokers and control group) (Zimmerman, 2004). The null hypothesis stated that there was no statistical difference between smokers and non-smokers. With an α-value set to 5 %, the null hypothesis was discarded if the significance level exceeded 95 %. After applying the Student-t-test to both datasets, a comparison was made between the manually and DL-based determined MLI values to determine if both approaches yielded similar conclusions. If they did, it could be inferred that the DL-based approach was a viable replacement for manual MLI.

The Student's t-test was used to test the hypothesis of a significant difference between the means of the two groups. In the t-test, the t-value was calculated by dividing the difference between the means by the standard error of the difference, and significance was determined by comparing the calculated t-value with the critical t-value at a predetermined significance level (^*^p = 0.05; ^**^p = 0.01; ^***^p = 0.001).

In summary, the implementation involved inducing COPD in mice through cigarette smoke exposure, followed by manual lung function assessment. *AIxCell* was employed to automate image analysis, utilizing a symmetrical convolutional encoder-decoder network, specifically the ICNet architecture, for semantic segmentation and MLI calculation. A pipeline comprising pre-processing, *AIxCell* modeling, and post-processing was established. Evaluation included calculating Intersection over Union (IoU) and F1-scores to assess segmentation accuracy, comparing MLI calculations between deep learning, classic segmentation, and manual labeling, and conducting Student's *t*-tests to evaluate differences in MLI between smokers and non-smokers.

## 3 Results

**Figure 4:**
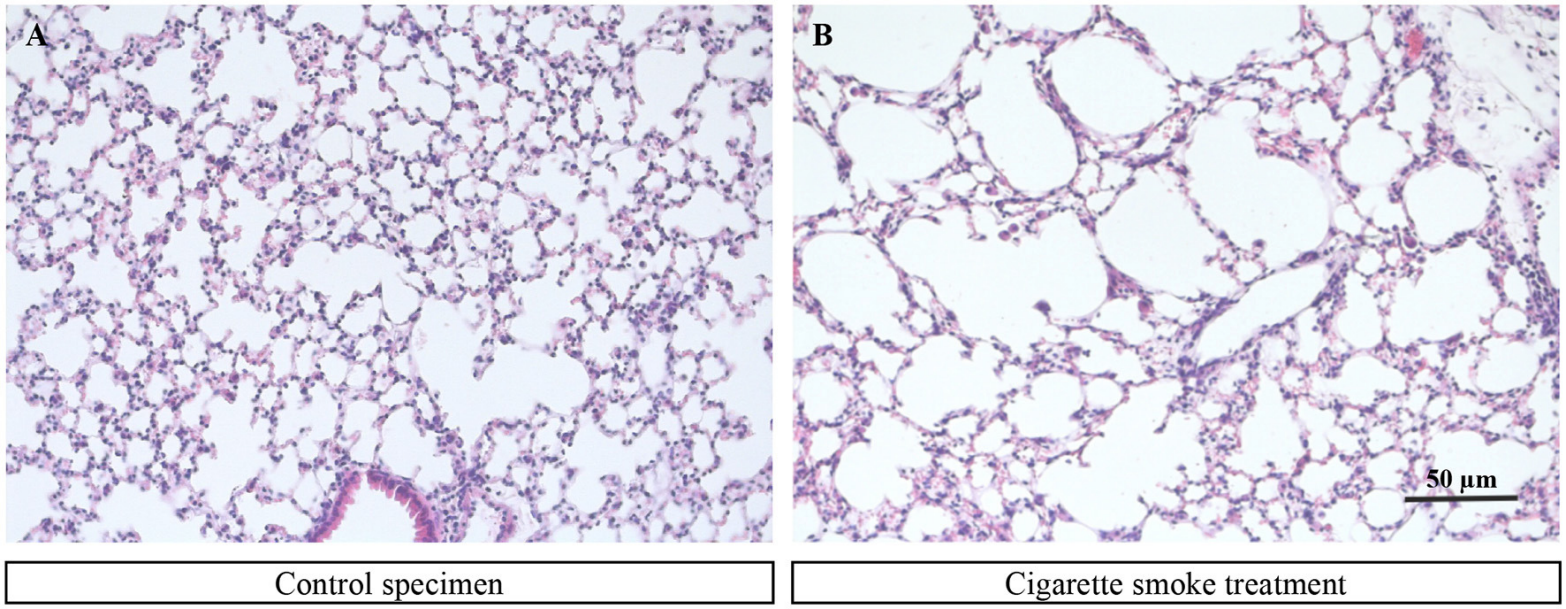
Exemplary images of a murine lung tissue sample from a control mouse **(A)** and a lung tissue sample from a mouse that was under the influence of cigarette smoke **(B)**.

In biological experiments, five months of exposure to smoke resulted in an inflammatory reaction in mouse lungs as the number of immune cells in bronchoalveolar lavage was strongly increased (Supplementary Figures S1A–C). This was accompanied by altered lung function in Flexivent measurements with elevated compliance as determined by snapshot pertubations at 50 mg/ml MCh (Supplementary Figure S1D) and therefore steeper pressure-volume (PV)- loops (Supplementary Figure S1E). Lung histology revealed airspace enlargement (Figure 4), which was quantified by quantifying MLI either manually or by automated approaches.

The MLI evaluation is based on lung tissue sections from control mice and mice exposed to cigarette smoke. Two of these colored tissue sections can be seen in Figure 4. The two tissue cross-section samples differed significantly in the size of the tissue lumens. Healthy lung tissue consists of smaller lumens that appear to break down due to excessive exposure to cigarette smoke, resulting in larger lumens. These pictures form the basis for manual evaluation and for evaluation using the DL pipeline.

The results of the DL-based based assessment of MLI can be divided into three subcategories: evaluation of training, validation, and test datasets, homogeneity of data in control- and smoker-group data sets, and finally the calculation of MLI values by automated and manual approaches. The evaluation of the ICNet training performance is based on the metrics IoU and F1 score. These are intended to provide information about the performance of object detection and accuracy. Afterwards, a comparison in the intersection count between manual and automated approaches is made to check the plausibility of used data and to determine potential differences. The last subsection evaluates the DL pipeline by comparing automated generated results with the data of the manual procedure.

### 3.1 Evaluation of training-, validation-, and test datasets

As already mentioned, the performance metrics IoU and F1-score are used to evaluate the accuracy of the segmentation model (ICNet). This is based on the classification of the training-, validation-, and test datasets into the categories: Tissue, Background, and Overall. The delineation of the categories is decided at the pixel level. The results of the two performance metrics of the selected CNN are shown in Table 1. Both the IoU values and the F1 scores show values above 90% for all categories (such as tissue or background) and form a reliable basis for the DL pipeline. On the left side of the table, three data sets (training, validation, and test) are listed. This is followed by the percentages of the three categories (tissue, background, and overall) for the IoU metric and then further to the right is the F1 score. The result of the applied IoU metric showed at least 92% in each category for all datasets, with the training dataset performing best with 93% or more in all categories. The accuracy calculated using the F1 metric showed a value between 96% and 97% for all datasets in all categories. The highest value which could be reached by using the F1 metric is 100%. The values from the F1 metric therefore indicated a high level of accuracy. The analysis of the training-, validation-, and test data showed that the percentage of false positives and false negatives was less than 8% and in some cases as low as 3%. These low values explain the robustness of the pipeline.

**Table 1:** IoU and F1-Score values with training, validation, and test data.

**Data set**	**IoU values**			**F1-Scores**		
	**Tissue**	**Background**	**Overall**	**Tissue**	**Background**	**Overall**
Training	0.93	0.94	0.93	0.97	0.97	0.97
Validation	0.92	0.92	0.92	0.96	0.96	0.96
Test	0.92	0.92	0.92	0.96	0.96	0.96

The underlying confusion matrix for determining the above performance metrics can be found in Table 2 for the test data set as an example. The values show that the system is very reliable and only in rare cases (1%–2%) wrong predictions were made. Predictions and annotations of tissue and background are thus juxtaposed. Predictions for tissue and background, which form a category, can be found in the left column of the table, and the top row shows the annotations for tissue and background, which together also form a category. The comparison of the two categories (prediction and annotation) shows the percentage of correct and incorrect assignments by the DL pipeline. The correct predictions are on the diagonal from left to right.

**Table 2:** Exemplary confusion matrix for determining IoU and F1-Score for the case of test data.

**Prediction**	**Annotation**			
		**Positive/tissue**	**Negative/background**	**Total**
	Positive/tissue	0.46	0.02	0.48
	Negative/background	0.01	0.51	0.52
	Total	0.47	0.53	1.00

### 3.2 Homogeneity of data in control- and smoker group data sets

To evaluate the DL pipeline, we analyzed the homogeneity within the groups (control/smoker), on the one hand for the manual process (manual cross testing) and on the other hand for the automated process (DL pipeline). Two hypotheses have been formulated to compare automated generated results with the data of the manual procedure. The hypotheses are defined below:

1. **H0-Null hypothesis:** The mean MLI of the two compared subjects is equal.
2. **H1-Alternative hypothesis:** The mean MLI of one subject is higher than that of the other.

First, the results of the manual cross test are shown, and then the results of the DL pipeline are highlighted. It should be noted that there are three control samples and three smoker samples compared to each other, resulting in three possible combinations, the resulting *p*-values are listed in (Table 3). It should be noted, that significances represent heterogeneity of the data. Homogeneity of the data is desirable in this experiment.

**Table 3:** DL-based cross testing did not show any difference within the smoker or control group.

**Sample set**	**Manual cross test**		**DL-Pipeline cross test**	
	**Control**	**Smoker**	**Control**	**Smoker**
Sample 1/2	0.064	0.414	0.553	0.937
Sample 1/3	0.018^*^	0.006^*^	0.962	0.674
Sample 2/3	0.510	0.029^*^	0.510	0.631

The manual and DL-based cross test was evaluated by comparing all *n* = 3 samples, within the smoker and control groups, by Student's *t*-test, table shows *p*-values, stars indicate significance.

Table 3 manual cross testing left table reveals that the manual process is more inconsistent than the DL-based cross test. Only in the cross tests of data obtained by the manual analysis there are significant differences within the control and smoker groups.

The statistical evaluation of the control group samples, which were determined by means of DL pipeline, does not show any significance. These findings reflect that the results of the DL pipeline are plausible and that the ICNet architecture used is suitable for automatic evaluation. Thus, both within the control group and within the smoker group, the null hypothesis could be confirmed. The different p-values resulting from the same samples, which were evaluated once automatically and once manually, result from the different evaluation approaches. The automated evaluation is the more direct measurement here, while the manual measurement is based purely on information about phase transitions.

In conclusion, no significant differences were found within the control or smoking groups by the uses of the DL pipeline. Thus, it can be stated that the smoking and control groups are homogeneous. Using the manual cross-test method, significances, i.e., differences within the groups, could be found more frequently.

### 3.3 Calculation of MLI values by automated and manual approaches

**Figure 5:**
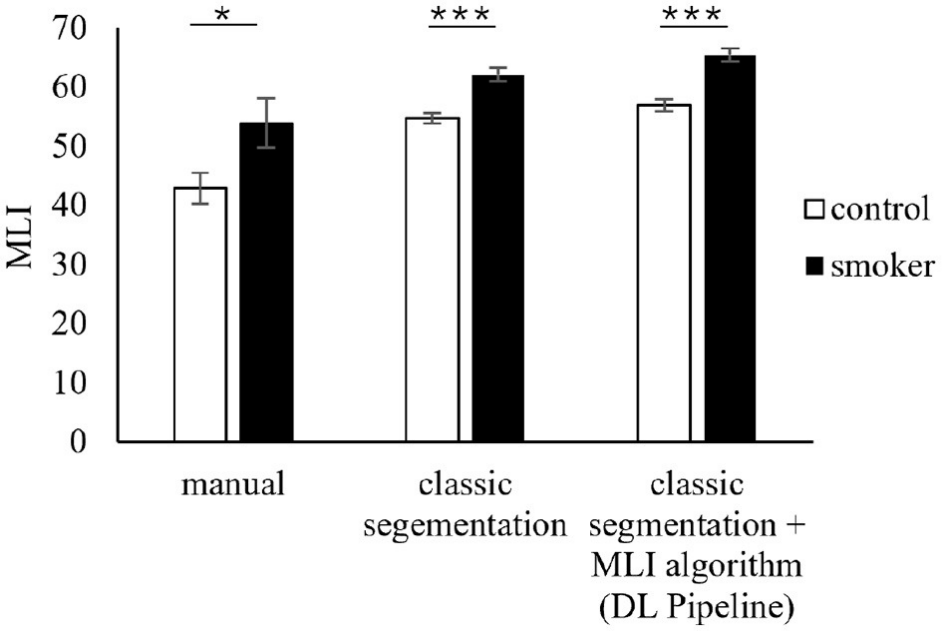
Comparison of the mean values of the two comparison groups (control group and smoker group) based on the MLI method calculated by manual evaluation, classical segmentation and DL segmentation.

After the homogeneity of the data could be ensured, the evaluation of the DL pipeline and the DL segmentation of the lung tissue sections were compared with the classic image segmentation and the manual analysis. When comparing the three segmentation approaches, it is noticeable that they are similar between the control group and the smoker group. The figure shows a significant difference between control and sample in each approach, as soon as image processing is added the significance level is raised from single significance to triple significance (Figure 5). In other words, the MLI values in the samples from the smoking group are higher than in the control group when using any segmentation method. This indicates that the chosen CNN provides valid results for the classification/segregation of the sample datasets. In addition, the scatter of the MLI results could be reduced, which is evident from the lower standard deviation of the control- and smoker group compared to the manual method.

The MLI values in the control and smoking groups are higher when using the DL pipeline and thus deviate from the data from the manual methods. A similar trend could also be observed when using the classic segmentation (Figure 5). The MLI values seem to vary between manual and automated approaches, which could be due to the different evaluation methodology. It is important to note that the ratio of control to sample is most significant here. As already seen in the homogeneity tests, manual and automated evaluations are not comparable in their absolute values, but this should not be the case, as the focus is on the difference between the samples to be compared, which should be similar for all three approaches.

In conclusion, the results of the DL pipeline provide a reliable and sufficient interpretation of lung tissue samples. Independent of the method, there is a clear difference between the MLI of the control and the smoker group. While the two automatic methods resulted in higher numbers for the MLI, their standard deviations were smaller. This may indicate a more consistent evaluation.

A student's *t*-test was executed to compare the three methods. No matter which method was used, the MLI of the smokers is significantly higher than the MLI of the control group. In addition, the *p*-value for all three methods was clearly lower than the significance level of 5%. Therefore, the currently available data suggests that the implementation of the DL-Pipeline is in fact functional. When comparing the time required for analysis, manual analysis is much more time consuming as, depending on the number of alveolar membrane cross sections, labeling and counting can take up to 10 min per image, which adds up to about 2h per mouse. In contrast, the AlxCell system can process the 12 images per mouse within few seconds.

## 4 Discussion

### 4.1 Model training and performance evaluation

#### 4.1.1 Model evaluation

The IoU scores of over 90 % throughout the data, shown in Section 3.1, indicate a good training performance. These high scores were expected due to the low complexity of the image segmentation task. This shows that the network architecture chosen by the AutoML algorithm was suitable for this application, demonstrating the usefulness of the *AIxCell* tool.

#### 4.1.2 Homogeneity of the data within the groups

The hypothesis tests confirmed that the DL model is superior to the manual approach in data consistency. In any case, the DL pipeline does not provide statistically relevant overlap between data of the same class (control or smoker). Therefore, our model can be considered a viable alternative to the manual approach and offers significant time savings. However, it is important to consider the limitation of the small sample size. To ensure a more reliable interpretation of the results, the collection of additional data is essential.

#### 4.1.3 Distinction between smokers and non smokers

Comparisons between the masks generated by classic segmentation and DL based segmentation using *AIxCell* show that the average MLI is slightly higher with *AIxCell's* approach, yet the difference is very small. The difference may be attributed to higher coarseness of the masks created by the DL algorithm. However, since the differences were generally very small, this can be seen as another proof of a good model performance.

Compared to the manually generated values, it becomes obvious that the two automatic approaches yielded notably larger values for the mean MLI (up to 33%), meaning they counted fewer intersections in each image than their human counterparts. Given that the DL model was trained using class segmentation data and closely adheres to its training data, the underlying reason can be attributed to the classical segmentation approach. This is because the result of a DL approach can only be as good as its training data (Alzubaidi et al., 2021).

Despite the apparent distinction between the manual and two automatic methods, notable differences between smokers and non-smokers exist across all scenarios. The mean MLI value serves as a reliable differentiation factor between these two groups and successfully fulfills its intended purpose in both instances. Consequently, it can be inferred that the *AIxCell* DL library is suitable for substituting or complementing manual analysis.

### 4.2 Potential for further improvement of the algorithm

Our results show that the DL-based image processing pipeline proposed by *AIxCell* is capable of automating MLI quantification in pulmonary emphysema analysis. However, different aspects can be taken into consideration for improving both accuracy and robustness of the analysis results. First, the relatively small dataset, consisting of 72 raw images underscores the need for additional data acquisition. This limitation arose from the restricted availability of biological samples. Nevertheless, the current dataset encompasses multiple lung sections from various individuals, thereby introducing variability between images. Even the small dataset already proved a general suitability of *AIxCell* for this application. Since oversampling and data augmentation has not been investigated in this research, besides collecting a higher quantity and more diverse genuine images from laboratory practice, we aim to investigate the impacts of data augmentation techniques (Leyendecker et al., 2023) on data diversity and model performance. Besides traditional data augmentation, generative adversarial networks (GANs) can be utilized for generating synthetic samples with the appearance of real images as proposed by Andreini et al. (2020). Chen et al. provide a review of GAN-based augmentation techniques in the medical domain (Chen et al., 2022). The analysis of the risk of overfitting, which is comparatively high for lab-specific cellular image analysis due to small dataset sizes and analysis-specific low diversity of the image data, will be enhanced by the increasing number of images. Training, validation, and pipeline testing become more extensive, increasing model robustness and accuracy. Additional genuine, augmented, and synthetically generated data will not only enable a more detailed analysis but also aims to mitigate overfitting in the first place by increasing data quantity and diversity (e.g., by geometrical transformations, color variation, and noise overlay).

Even though we consider data-centric improvement potentials much larger compared to model-centric ones, new network architectures for semantic image segmentation like attention-based transformers can be taken into account (Strudel et al., 2021). Although we contend that converting from three color channels to grayscale results in minimal information loss due to the distinct contrast between tissue and background, this topic could be explored in future research. For post-processing, we consider the proposed MLI algorithm as a fast and reliable solution. However, a more extensive parameter study on historical and manually analyzed data could help align automatic MLI quantification with human results and thus increase the comparability of scientific results. Due to the specificity of counting intersections, this task cannot be automatically applied to other *AIxCell* use cases, unlike the segmentation component. As a result, *AIxCell*'s core functionalities remain focused on segmentation. Future evaluations of additional use cases could further demonstrate *AIxCell*'s versatility. It has been successfully used to classify cardiomyocyte images (Leyendecker et al., 2022), and has also been applied to Embryoid Bodies, induced pluripotent stem cells, and mesenchymal stem cells. In these applications, segmentation was achieved with *AIxCell*'s autoML algorithm, while both pre-processing and post-processing were tailored to specific tasks.

### 4.3 Comparison and summary

Manual MLI quantification is a widespread and accepted method for analyzing airspace enlargement in COPD research. First approaches to automate the task have already been made in the 1970s when computational power was still limited, emphasizing the importance of this task. Langston et al. compared the average *L*_*m*_ between human and machine measurement. Similar to this paper, they found that *L*_*m*_ created by humans and machines strongly correlate with each other, even if specific values differ. Thus, the computational model can be used to replace human *L*_*m*_ measurement. Crowley et al. (2019) use a semi-automated approach that solely relies on openly available software. Their segmentation algorithm uses traditional thresholding methods instead of neural networks. The results are equally good as in this study. However, *AIxCell* aims for a fully-automated solution to take away even more work from the user. Other studies replace MLI with area based methods, where the area of cavities is calculated computationally (Knudsen et al., 2010).

Due to the continued use of MLI despite the possibilities to use new approaches caused by increased computational resources, the results can also be compared with evaluations carried out years ago. However, it has to be said that MLI is certainly not the most precise way to analyze lung tissue and only allows random sample testing (Salaets et al., 2020). Before computers were widely available and powerful, a simple method that could also be manually carried out must have been used (Liu et al., 2017). With modern computational capacities though, the volume of cavities can be determined easily with little more effort than MLI (Sallon et al., 2015). Other optical methods for automatic lung disease detection have already been proposed (Gupta et al., 2019). Therefore, a switch to a more precise method could pay out long term. The biological limitations of this approach further include the potential dependency of emphysema severity on perfusion pressure and lung inflation during tissue processing. In addition, using two-dimensional mouse lung sections may not accurately represent the three-dimensional in vivo conditions in a living animal.

In conclusion, this study presented an automated DL-based approach to MLI quantification in microscopy images of histological lung sections. Even though the considered analysis task does not require a DL-based approach for image segmentation in preparation to MLI computation, the results show the functionality and widespread applicability of *AIxCell*. By successfully automating MLI quantification, our approach enhances efficiency, reliability, and objectivity of emphysema quantification in COPD research and relives biomedical experts of the cumbersome task of manual MLI determination.

## Data Availability

The data analyzed in this study is subject to the following licenses/restrictions: the data set used is the property of Department of Systems Physiology, Medical Faculty, Ruhr University of Bochum and Institute of Physiology I, Medical Faculty, University of Bonn. Data access and further information on the data can be requested from corresponding authors. Requests to access these datasets should be directed to Daniela Wenzel, daniela.wenzel@rub.de.

## Supplementary material

Supplementary Figure 1 Model of cigarette smoke-induced COPD. **(A, B)** Cells in bronchoalveolar lavage (BAL) fluid after Diff-Quick staining of controls **(A)** and mice exposed to cigarette smoke (CS) for 5 months **(B)**, scale bar: 20 μm. **(C)** Absolute cell numbers of macrophages (Mac), neutrophils (Neu), and lymphocytes (Lym) in BAL. **(D)** Lung compliance in response to methacholine inhalation (50 mg/ml). **(E)** Pressure volume loops in control mice and animals exposed to CS, ^*^p < 0.05, ^*^p < 0.01.
