# Supplementary figures and images for: Deep learning based automation of mean linear intercept quantification in COPD research
Source: Front Big Data. 2025 Jun 10;8:1461016. doi: 10.3389/fdata.2025.1461016 (PMC12186305; doi:10.3389/fdata.2025.1461016)

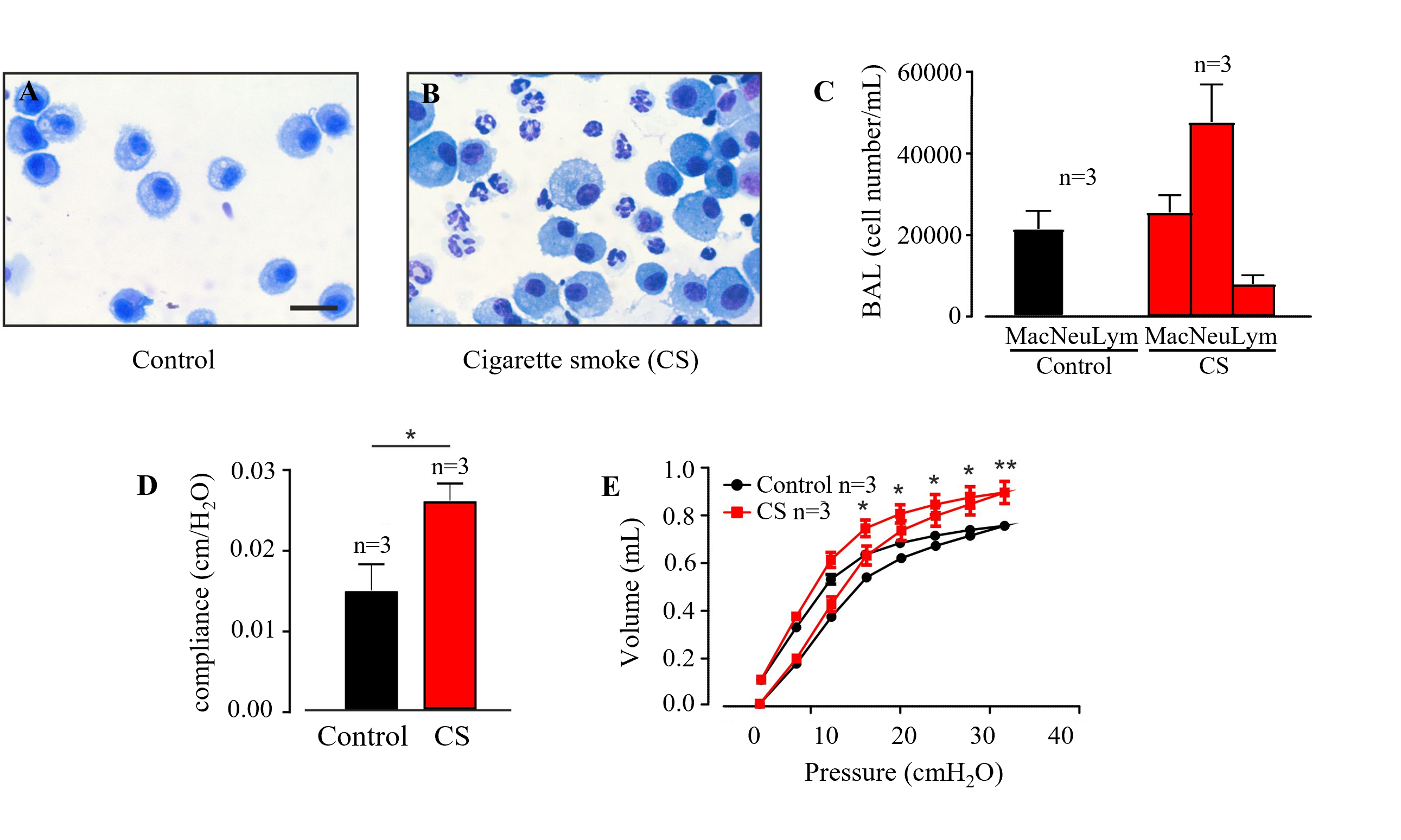

Supplement: Supplementary Figure 1 — Model of cigarette smoke-induced COPD. (A, B) Cells in bronchoalveolar lavage (BAL) fluid after Diff-Quick staining of controls (A) and mice exposed to cigarette smoke (CS) for 5 months (B), scale bar: 20 μm. (C) Absolute cell numbers of macrophages (Mac), neutrophils (Neu), and lymphocytes (Lym) in BAL. (D) Lung compliance in response to methacholine inhalation (50 mg/ml). (E) Pressure volume loops in control mice and animals exposed to CS, *p < 0.05, *p < 0.01. [file Image_1.png]
